# Case Report: Robust and durable response to the combination of tislelizumab and chemotherapy in advanced thymic epithelial tumors: a case series

**DOI:** 10.3389/fimmu.2025.1516297

**Published:** 2025-05-27

**Authors:** Lantian Zhang, Yuqi Zhang, Shuke Li, Yan Wang, Yue Yu, Jing He, Wen Gao

**Affiliations:** ^1^ Department of Oncology, The First Affiliated Hospital of Nanjing Medical University, Nanjing, China; ^2^ Endoscopy Center, The First Affiliated Hospital of Nanjing Medical University, Nanjing, China; ^3^ The Friendship Hospital of Ili Kazakh Autonomous Prefecture, Ili & Jiangsu Joint Institute of Health, Yining, China; ^4^ Department of Thoracic Surgery, The First Affiliated Hospital of Nanjing Medical University, Nanjing, China

**Keywords:** tislelizumab, immunotherapy, thymoma, thymic carcinoma, first-line treatment, case report

## Abstract

**Background:**

Thymic epithelial tumors (TETs), categorized predominantly as thymoma (T) or thymic carcinoma (TC), face a challenging prognosis and limited treatment options. Although chemotherapy remains the established treatment for advanced TETs, its responses tend to be short-lived. The emergence of immunotherapy, particularly programmed cell death-1 (PD-1) and programmed death ligand-1 inhibitors (PD-L1), is increasingly being regarded as a promising new treatment option for various malignancies.

**Methods:**

Herein, we present a case series of eight patients with TETs who received tislelizumab treatment at Jiangsu Provincial Hospital between 2021 and 2023. All cases were histologically confirmed as either thymoma or thymic carcinoma. Among these eight cases, six patients (5 thymic carcinomas [TC] and 1 thymoma [T]) received tislelizumab in combination with chemotherapy following multiple cycles of prior chemotherapy without achieving significant therapeutic response. Two TC patients were administered this combination regimen as first-line treatment. Following the initiation of immunotherapy, patients received tislelizumab at a dose of 200 mg every three weeks until disease progression or the occurrence of unacceptable toxicity. Treatment response was assessed by the investigators according to the Response Evaluation Criteria in Solid Tumors (RECIST) version 1.1 guidelines.

**Results:**

The 8 patients described had a median age of 59 years (range, 47-72). During the course of immunotherapy, five patients (62.5%) achieved partial response, and notably, even after transitioning to maintenance therapy with tislelizumab, the lesions continued to shrink, with the longest sustained partial response lasting over 2 years. Three patient (37.5%) experienced stable disease as their best response to immunotherapy. Among all these patients, three patients (37.5%) demonstrated initial efficacy but subsequently exhibited progressive disease (median progression-free survival of 14 months). All patients are still being followed up, with the longest PFS extending to 31 months. Notably, five of the eight patients underwent PD-L1 testing and were all found to be negative. Despite this, no immune-related Grade 3–5 adverse events (AEs) were reported and all AEs were manageable with supportive measures. Grade 1–2 AEs were adrenal insufficiency (n=1), thyroid dysfunction (n=1), and pneumonia (n=1).

**Conclusions:**

Our study findings suggest that the combination of immunotherapy and chemotherapy yields durable clinical responses in patients with TETs, suggesting its potential as a safe and effective first-line treatment strategy for advanced TETs. Notably, the therapeutic benefits of chemo-immunotherapy appear to extend beyond patients with high PD-L1 expression (≥50%), indicating that this treatment approach may not be strictly limited to individuals with elevated PD-L1 levels.

## Introduction

Thymic epithelial tumors (TETs) are a common type of anterior mediastinal tumor, including thymoma and thymic carcinoma (1, 2). The incidence of TETs ranges from 1.3 to 3.2 cases per million and is on the rise annually (3). The World Health Organization (WHO) classifies these tumors into thymoma types A, AB, B1, B2, B3, and thymic carcinoma (4). Thymic carcinoma represents only 0.06% of all TETs and is more likely to metastasize distantly compared to thymoma (5). For advanced tumors, platinum-based regimens have become the standard treatment but the efficacy remains limited (6). The response rate for first-line treatment in advanced thymoma (cyclophosphamide, doxorubicin, and cisplatin) is approximately 44% (7). For advanced thymic carcinoma, the response rate to first-line treatment (carboplatin and paclitaxel) ranges from 22% to 36% (8–11). Targeted therapies and anti-angiogenics are also limited, with no regimen demonstrating consistent benefit (12–14).

The thymus is a crucial immune organ where programmed death-ligand 1 (PD-L1) is abundantly expressed in TETs, with positivity rates ranging from 23% to 68% in thymomas and 36% to 80% in thymic carcinomas (15–17). Notably, PD-L1 has been established as a predictive biomarker for immunotherapy response in TETs. Clinicopathological analyses further reveal that high PD-1/PD-L1 expression correlates with more aggressive tumor characteristics and serves as an independent prognostic factor associated with significantly poorer clinical outcomes (18, 19). These collective findings suggest that immune checkpoint inhibitors (ICIs) targeting the PD-1/PD-L1 axis may represent a promising therapeutic strategy for TETs. Completed clinical trials have demonstrated encouraging efficacy of immunotherapy as a second-line treatment for TETs. For instance, a phase II trial by Cho et al. (20) evaluated pembrolizumab in 33 patients (7 with thymoma and 26 with thymic carcinoma), reporting an overall response rate (ORR) of 21.2%, a disease control rate (DCR) of 78.8%, and a median progression-free survival (PFS) of 6.1 months.

However, immune-related adverse reactions have drawn attention. In the phase 2 trial conducted by Giaccone et al. (21), every patient experienced immune-related adverse events, with six patients developing severe complications including myocarditis and hyperglycemia. Due to differences in the immune microenvironment between thymoma and thymic carcinoma, the incidence of immune-related adverse reactions varies significantly. In the study by Cho et al. (20), these adverse events were observed in 15.4% of thymic carcinoma patients but in 71.6% of thymoma patients, suggesting a higher safety profile for immunotherapy in thymic carcinoma. Additionally, research has highlighted unique, high-grade immune-related adverse reactions rarely seen in other tumor types, such as myocarditis, myositis, and severe myasthenia, in thymic tumors (22, 23). Based on this evidence, the National Comprehensive Cancer Network (NCCN) recommends pembrolizumab monotherapy as a second-line treatment for thymic carcinoma (24). Notably, the occurrence of immune-related adverse events has been associated with better treatment outcomes. Giaccone et al. (21) found that 4 out of 9 patients with severe complications achieved partial responses, a significantly higher response rate compared to patients without such events. These findings underscore the need to develop safer and more effective therapies for thymic epithelial tumors.

Tislelizumab is a novel monoclonal antibody developed in China that targets programmed cell death-1 (PD-1). It has been specifically engineered to minimize binding to Fc gamma receptors (FcγR) on macrophages, thereby reducing the risk of antibody-dependent phagocytosis (25). Clinical applications have demonstrated its efficacy in various solid tumors, including lung cancer, gastric cancer, and esophageal cancer (26, 27). Studies have shown that the structural modification of tislelizumab may lower the incidence of severe immune-related adverse events (irAEs) (28, 29). Furthermore, evidence supports that combining immunotherapy with standard chemotherapy offers synergistic benefits compared to chemotherapy alone. This approach overcomes the limitations of PD-(L)1 inhibitor monotherapy, which is often restricted by PD-L1 expression levels, thereby enhancing therapeutic efficacy (30–33).

To the best of our knowledge, there are no published data supporting the use of tislelizumab plus chemotherapy in advanced TETs. To address this gap, we present a case series involving eight patients with advanced TETs (**Table 1**). These patients were categorized according to whether they received tislelizumab as their initial treatment, and informed consent was obtained from all participants prior to their inclusion in the study.

**Table 1:** Baseline characteristics.

Case	Gender/age	Masaoka-Koga Stage	Histology	Metastasis	Baseline Tumor size (cm)	PD-L1 TPS, %
**1**	Female/49y	IV	TC	Mediastinum, Lungs, Lymph node	3.6×2.2	/
**2**	Male/47y	IV	T(B3)	Mediastinum, Pleura	6.75×2.7	/
**3**	Male/60y	IV	TC	Mediastinum, Lungs, Pleura, lymph node	3.7×2.9	<1
**4**	Male/70y	IV	TC	Mediastinum, Pericardiac fluid, Lymph node	12.3×9.8	<1
**5**	Male/72y	IV	TC	Mediastinum, Pericardiac fluid	7.4×4.3	<1
**6**	Male/58y	IV	TC	Mediastinum, Lungs, Pleura	5.7×4.1	<1
**7**	Male/60y	IV	TC	Mediastinum, Lungs, Liver	6.7×2.8	<1
**8**	Male/58y	IV	TC	Mediastinum, Lymph node	5.2×3.6	/

Date of date cut-off: July 2024; TC, thymic carcinoma; T, thymoma; PD-L1, programmed death ligand-1; TPS, tumor cell proportion score.

## Methods

### Patient characteristics

This study analyzed 8 patients with advanced unresectable or metastatic thymic tumors (7 patients with TC and 1 patient with T) who received at least 2 cycles of tislelizumab immunotherapy at the First Affiliated Hospital of Nanjing Medical University between 2021 and 2023. The median age was 59 years (range, 47-72). All patients had an Eastern Cooperative Oncology Group (ECOG) performance status of <2 at treatment initiation. All tumor staging in this study was based on the Masaoka-Koga staging criteria. The cohort comprised 8 stage IV thymic tumor patients, including 4 cases with lung metastases and 2 cases with pericardial effusion. All enrolled patients were treatment-naïve and did not receive concurrent anti-angiogenic therapy or radiotherapy.

### Treatment rationale

Among the 8 patients in this case series, 6 received chemotherapy as first-line treatment. The treatment regimens were stratified by histological subtype. One thymoma patient treated with cyclophosphamide (500 mg/moph day 1), doxorubicin (50 mg/m²on day 1), and cisplatin (50 mg/mlat day 1), and five thymic carcinoma patients who received either paclitaxel (200 mg/mi on day 1) or paclitaxel liposome (135 mg/msom day 1) plus carboplatin (AUC=5 on day 1). The specific treatment plan for each case can be found in **Table 2**.

**Table 2:** Treatment regimens and prognostic information.

Case	Treatment regimen	Best responses	PFS (month)
1	C1-C2: paclitaxel liposome and carboplatin on Day 1 C3-C8: tislelizumab (200 mg), paclitaxel liposome and carboplatin on Day 1 C9-16: tislelizumab 200 mg every 3 weeks	PR	31
2	C1-C3: doxorubicin, cyclophosphamide and cisplatin on Day 1 C4-C6: tislelizumab (200 mg), doxorubicin, cyclophosphamide and cisplatin on Day 1 C7-15: tislelizumab 200 mg every 3 weeks	PR	NR
3	C1-C2: paclitaxel liposome and carboplatin on Day 1 C3-C6: tislelizumab (200 mg), paclitaxel liposome and carboplatin on Day 1 C7-C12: tislelizumab 200 mg every 3 weeks	PR	NR
4	C1-C2: paclitaxel and carboplatin on Day 1 C3-C6: tislelizumab (200 mg), paclitaxel and carboplatin on Day 1 C7: tislelizumab 200 mg every 3 weeks After C7: radiation therapy	SD	NR
5	C1: paclitaxel and carboplatin on Day 1 C2-C6: tislelizumab (200 mg), paclitaxel and carboplatin on Day 1 After C6: radiation therapy C7: tislelizumab 200 mg every 3 weeks	SD	NR
6	C1-C2: paclitaxel liposome and carboplatin on Day 1 C3-C6: tislelizumab (200 mg), paclitaxel liposome and carboplatin on Day 1	SD	6
7	C1-C6: tislelizumab (200 mg), paclitaxel liposome and carboplatin on Day 1 C7-C10: tislelizumab 200 mg every 3 weeks	PR	14
8	C1-C6: tislelizumab (200 mg), paclitaxel and carboplatin on Day 1 C7-C18: tislelizumab 200 mg every 3 weeks	PR	NR

C, cycle; PR, partial response; PD, progressive disease; SD, stable disease; NR, not reached; PFS, progression-free survival.

Due to suboptimal response, immunotherapy was subsequently added. Although anti-PD-1 antibody therapy for thymic tumors has not yet been approved by the U.S. Food and Drug Administration (FDA), we incorporated immunotherapy based on emerging evidence suggesting its potential efficacy in thymic tumors, despite the recognized risk of irAEs.

In our pursuit of safe and effective immunotherapeutic strategies, tislelizumab was selected based on clinical experience and its favorable safety profile. Chemotherapy was discontinued after 4–6 cycles, and patients achieving disease stabilization or partial response continued tislelizumab as maintenance therapy until unacceptable toxicity, disease progression, or death. Toxicity was evaluated using the Common Terminology Criteria for Adverse Events (CTCAE) version 5.0, with dose adjustments guided by clinical protocols. The immune-related adverse events observed in our case series are detailed in **Table 3**. Treatment efficacy was assessed by investigators according to the Response Evaluation Criteria in Solid Tumors (RECIST) version 1.1.

**Table 3:** Immune-related adverse events in the tislelizumab therapeutic course (graded by CTCAE 5.0).

Case	Adverse effect	Maximum Grade	Duration	Treatment	Prognosis
1	Immune-related pneumonia	2	Cycle14-Cycle16	Hormone therapy	Well managed
2	Immune-related adrenal insufficiency	2	Cycle5-Cycle7	Systemic corticosteroid therapy	Well managed
5	Immune-related thyroid dysfunction	2	Before Cycle5	Suspension of immunotherapy	Well managed
			Cycle6-Cycle7	Thyroid hormone therapy	

## Case reports

### Cases 1 to 6—Immunotherapy used following several cycles of chemotherapy without significant efficacy

#### Case 1

**Figure 1:**
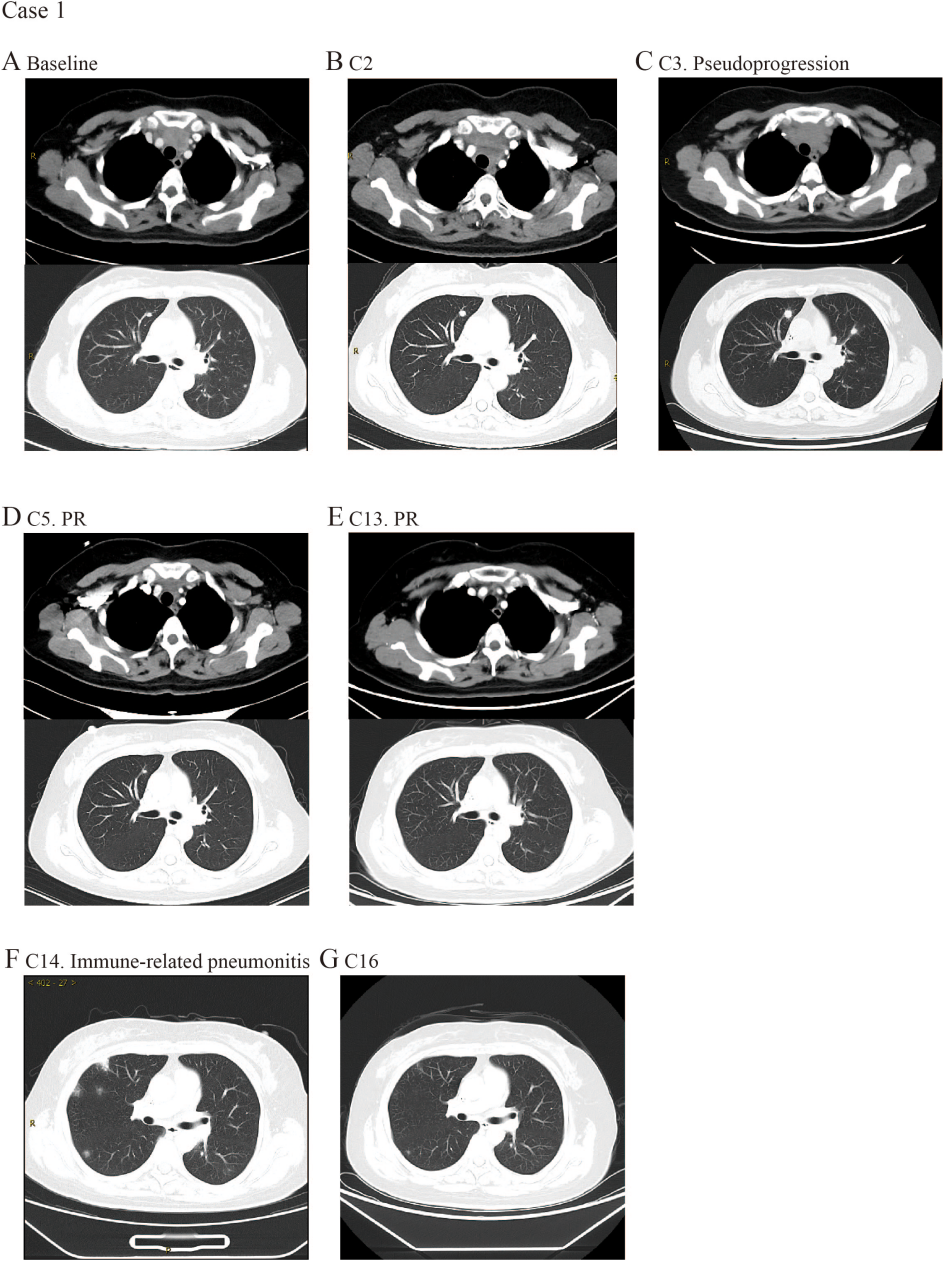
CT images selected from case 1. **(A)** CT scan examination of the primary tumor at the first time of diagnosis. **(B)** CT scan examination of the tumor after 2 cycles of treatment. **(C)** Pseudoprogression following the initial cycle of combination therapy. **(D)** The primary tumor size decreased after 5 cycles of treatment. **(E)** The primary tumor size decreased after 13 cycles of treatment. **(F)** CT scan performed after 14 cycles of treatment revealed new small patches of ground-glass opacity and areas of high density in both lungs, indicating the possibility of immune-related pneumonia. **(G)** Immune-related pneumonia was well managed after hormone therapy.

A 49-year-old female presented with throat pain and cough and was diagnosed with stage IV TC in September 2021. A chest computed tomography (CT) scan revealed a soft tissue mass measuring 3.6×2.2 cm in the anterior superior mediastinum, along with multiple lung nodules and left hilar lymphadenopathy (**Figure 1A**). The PD-L1 expression level was not measured. As the initial treatment, she received a first-line chemotherapy regimen consisting of paclitaxel liposome and carboplatin. After undergoing two cycles of this chemotherapy, the patient exhibited stable disease (SD). However, both the tumor and pulmonary nodules increased in size (**Figure 1B**). In consideration of her age, the swift onset of symptoms and the existing evidence regarding the individual effectiveness of chemotherapy and anti-PD-1 therapy for TETs, immunotherapy combined with chemotherapy was recommended. The patient was then started on tislelizumab at a dosage of 200 mg every 3 weeks, in conjunction with the chemotherapy regimen mentioned above (paclitaxel liposome and carboplatin) from November 2021. According to the overall situation of the patient during treatment, progressive disease (PD) that occurred following the initial cycle of combination therapy was considered as pseudoprogression (**Figure 1C**). CT scan showed the marked shrinkage of the anterior mediastinal tumor (partial response, PR) after 5 cycles of combination therapy (**Figure 1D**). Therapy was modified to maintenance therapy with tislelizumab alone at a dosage of 200 mg every 3 weeks from May 2022. Subsequent imaging studies indicated further reduction in tumor size during the maintenance phase (**Figure 1E**). Unfortunately, disease progression occurred after 23 months of maintenance therapy, characterized by lymph node lesion enlargement. Finally, the patient experienced PD and achieved a progression-free survival (PFS) duration of 31 months. Notably, the patient developed grade 2 immune-related pneumonia (irP) before the 7th cycle of maintenance immunotherapy. A June 2023 CT scan revealed new bilateral ground-glass opacities and consolidations, consistent with grade 2 immune-mediated pneumonitis (CTCAE 5.0) (**Figure 1F**). Given the patient’s absence of symptoms and normal oxygen saturation, corticosteroid therapy was initiated while immunotherapy was continued. A follow-up CT scan prior to the 9th maintenance immunotherapy cycle showed reduction in the multifocal patchy opacities and consolidations in both lungs (**Figure 1G**). The irP remained manageable with supportive measures.

#### Case 2

A 47-year-old man was diagnosed with stage IV B3-type thymoma according to the WHO classification in November 2022. CT revealed a soft tissue mass measuring 6.75×2.7cm in the anterior mediastinum, accompanied by nodular thickening in the left pleura, which suggested possible invasive thymoma with pleural metastasis. The PD-L1 expression level was not measured. Chemotherapy consisting of liposomal doxorubicin, cyclophosphamide and cisplatin was introduced as first-line therapy since November 2022. Each 3-week cycle consisted of doxorubicin, cyclophosphamide and cisplatin on Day 1. However, after 3 cycles, the patient exhibited SD with no significant change in tumor size. Considering the patient had no paraneoplastic manifestations of his TET, the regimen was adjusted to tislelizumab at a dosage of 200 mg every 3 weeks alongside the previously mentioned chemotherapy regimen from February 2023. Each 3-week cycle consisted of tislelizumab (200 mg), doxorubicin, cyclophosphamide and cisplatin on Day 1. After 3 cycles of combination treatment, a notable reduction in the size of the anterior mediastinal tumor was observed, indicating a partial response (PR). Treatment was switched to tislelizumab monotherapy at a dosage of 200 mg every 3 weeks since May 2023. Maintenance therapy was continued up to 14 months due to a good response. The most recent CT revealed a significantly reduced tumor size of 3.3×3.2 cm. The PFS of this patient has been extended to about 20 months so far. Notably, after the first cycle of immunotherapy, the patient’s cortisol level decreased by 40% from baseline, likely immunotherapy-related, leading to a diagnosis of grade 2 adrenal insufficiency (CTCAE 5.0). In May 2023, the patient was initiated on systemic corticosteroid therapy. The patient received hormone replacement therapy until September 2023 and responded well to corticosteroid treatment. No other new autoimmune disorders were observed.

#### Case 3

**Figure 2:**
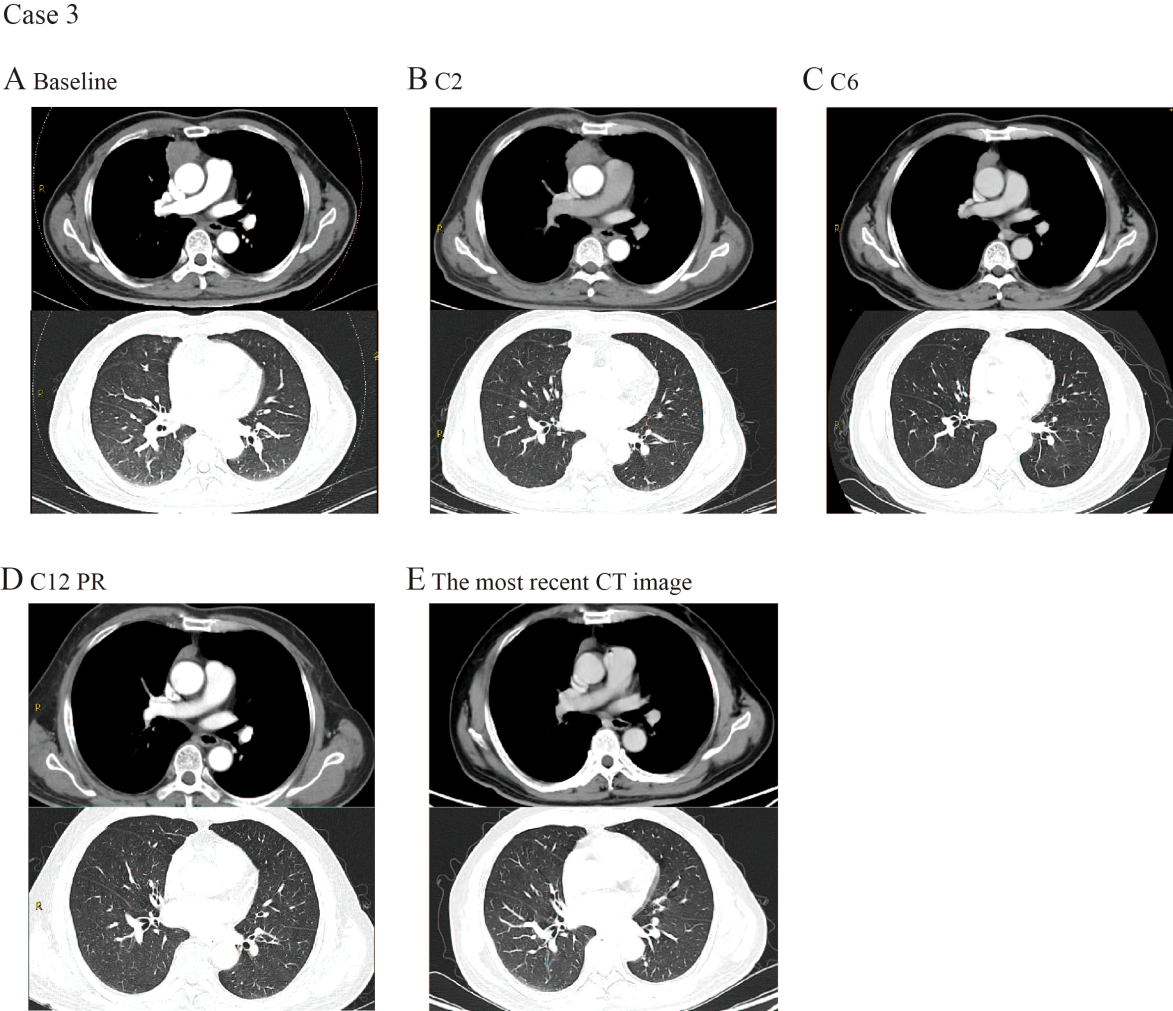
CT images selected from case 3. **(A)** CT scan examination of the primary tumor at the first time of diagnosis. **(B)** CT scan examination of the tumor after 2 cycles of treatment. **(C)** The primary tumor size decreased after 6 cycles of treatment. **(D)** The primary tumor size decreased after 12 cycles of treatment. **(E)** The most recent CT image.

A 60-year-old male was diagnosed with stage IV TC in August 2023. CT revealed a mediastinal mass measuring 3.7×2.9 cm, along with multiple lung nodules and multiple nodular shadows along the pleura (**Figure 2A**). The PD-L1 expression level was found to be less than 1%, indicating a potentially limited response to immunotherapy. Initially, the patient received a standard chemotherapy regimen comprising paclitaxel liposome and carboplatin. After 2 cycles of chemotherapy, a SD was observed (**Figure 2B**). Beginning in October 2023, the treatment regimen was adjusted to tislelizumab combined with chemotherapy for a total of four cycles. This sequential approach proved beneficial, resulting in a sustained partial response (PR) (**Figure 2C**). The treatment was changed to tislelizumab monotherapy at a dosage of 200 mg every 3 weeks in February 2024 and continued for up to 6 cycles (**Figure 2D**). Remarkably, the patient achieved lasting remission despite a low PD-L1 expression rate of less than 1% according to the tumor proportion score (TPS). The latest CT scan showed that the pleural nodules were not clearly visible (**Figure 2E**). The PFS of this patient has been extended to about 11 months so far. Throughout the course of these combination treatments, no serious adverse events were observed.

#### Case 4

A 70-year-old male was diagnosed with stage IV TC with malignant pericardial effusion in July 2023. A CT scan revealed a sizable mediastinal mass measuring approximately 12.3×9.8 cm and Ultrasonographic scan showed massive pericardiac fluid. The PD-L1 expression level was found to be less than 1%. The patient received paclitaxel plus carboplatin as the first-line treatment in August 2023. Each 3-week cycle consisted of paclitaxel and carboplatin on Day 1. However, the initial results of this regimen showed minimal improvement in his condition. After 2 cycles of chemotherapy, the patient was treated with 4 cycles of tislelizumab plus chemotherapy since October 2023 to enhance the treatment efficacy. Each 3-week cycle consisted of tislelizumab (200 mg), paclitaxel and carboplatin on Day 1. Tislelizumab monotherapy at a dosage of 200 mg every 3 weeks was continued up to 1 cycle. The efficacy based on the measured tumor sizes was still evaluated as SD. Considering the significant improvement in the patient’s pericardial effusion, radiation therapy was initiated from March 2024 to April 2024. The latest CT scan showed that the primary tumor has shrunk to 9.6×8.7cm. The PFS of this patient has been extended to about 11 months so far. The patient experienced no obvious discomfort during the treatment.

#### Case 5

A 72-year-old male presented chest tightness was diagnosed with stage IV TC with supraclavicular and mediastinal lymph node metastasis in July 2023. A CT scan revealed a soft tissue mass in the anterior mediastinum measuring roughly 7.3×4.3 cm, accompanied by minor pleural and pericardium effusion. The PD-L1 expression level was found to be less than 1%. A first line was initiated in July 2023 with chemotherapy (paclitaxel and carboplatin) showing poor efficacy with significantly increased pericardial effusion compared with before. Each 3-week cycle consisted of paclitaxel and carboplatin on Day 1. To alleviate the symptoms of compression, pericardiocentesis and drainage were performed. The patient was then started on tislelizumab at a dosage of 200 mg every 3 weeks combined with the chemotherapy mentioned above. Following 3 cycles of combination treatment, a SD was noted with a reduction in the anterior mediastinal mass and significantly reduced pericardial effusion. It is noteworthy that prior to the fourth cycle of immunotherapy combined with chemotherapy in November 2023, the patient developed thyroid dysfunction, manifested by a decreased thyroid-stimulating hormone (TSH) level, consistent with Grade 2 immune-related thyroid dysfunction (CTCAE 5.0). Immunotherapy was withheld during this cycle. Subsequent pre-treatment evaluations in later cycles showed normalized thyroid function, prompting the resumption of the combination therapy. Later, in January 2024, the patient experienced another episode of decreased TSH levels, leading to the initiation of thyroid hormone therapy. The patient responded well to hormone therapy. To enhance efficacy, radiation therapy was administered from February to March 2024, followed by one cycle of tislelizumab monotherapy. The most recent CT scan revealed that the primary tumor has decreased in size to 5.4×1.5 cm. The PFS of this patient has been extended to about 12 months so far.

#### Case 6

A 58-year-old male presented with retrosternal pain and was diagnosed with stage IV TC in February 2023. A CT scan revealed a mass measuring 5.7×4.1 cm in the anterior mediastinum, as well as enlarged lymph nodes in both lungs and metastasis to the left pleura. The PD-L1 expression level was found to be less than 1%. The patient was treated with a first-line regimen consisting of paclitaxel liposome and carboplatin. Each 3-week cycle consisted of paclitaxel liposome and carboplatin on Day 1. After 2 cycles of chemotherapy, a SD was observed. the regimen was adjusted to tislelizumab at a dosage of 200 mg every 3 weeks combined with the chemotherapy mentioned above since April 2023. Later disease progression resulted in treatment cessation after 4 cycles of combination therapy, characterized by the increased tumor size and nodule of the chest wall. The patient resulted in PD with a PFS time of 6 months. The patient experienced no immune-related adverse events during the treatment.

### Cases 7 to 8 —Immunotherapy combined with chemotherapy as the first-line treatment

#### Case 7

A 60-year-old male presented with retrosternal pain and received a diagnosis of stage IV TC in April 2023. A CT scan identified a soft tissue mass measuring 6.7suri cm located in the anterior superior mediastinum, along with suspected liver metastases. The PD-L1 expression level was found to be less than 1%. It is noteworthy that even patients with negative or low PD-L1 levels can still achieve durable responses to immunotherapy. The patient received tislelizumab combined with chemotherapy (paclitaxel liposome and carboplatin) as a first-line treatment from April 2023. Following six cycles of this combination treatment, a PR was noted, characterized by a reduction in both the tumor size and liver metastases. The treatment was switched to tislelizumab monotherapy at a dosage of 200 mg every 3 weeks in November 2023. To improve efficacy, the patient also accepted percutaneous radiofrequency ablation of residual liver metastasis in January 2024. Unfortunately, disease progression occurred in June 2024 after 4 cycles of tislelizumab monotherapy, resulting in PD with a PFS time of 14 months. The patient experienced no immune-related adverse events during the treatment.

#### Case 8

**Figure 3:**
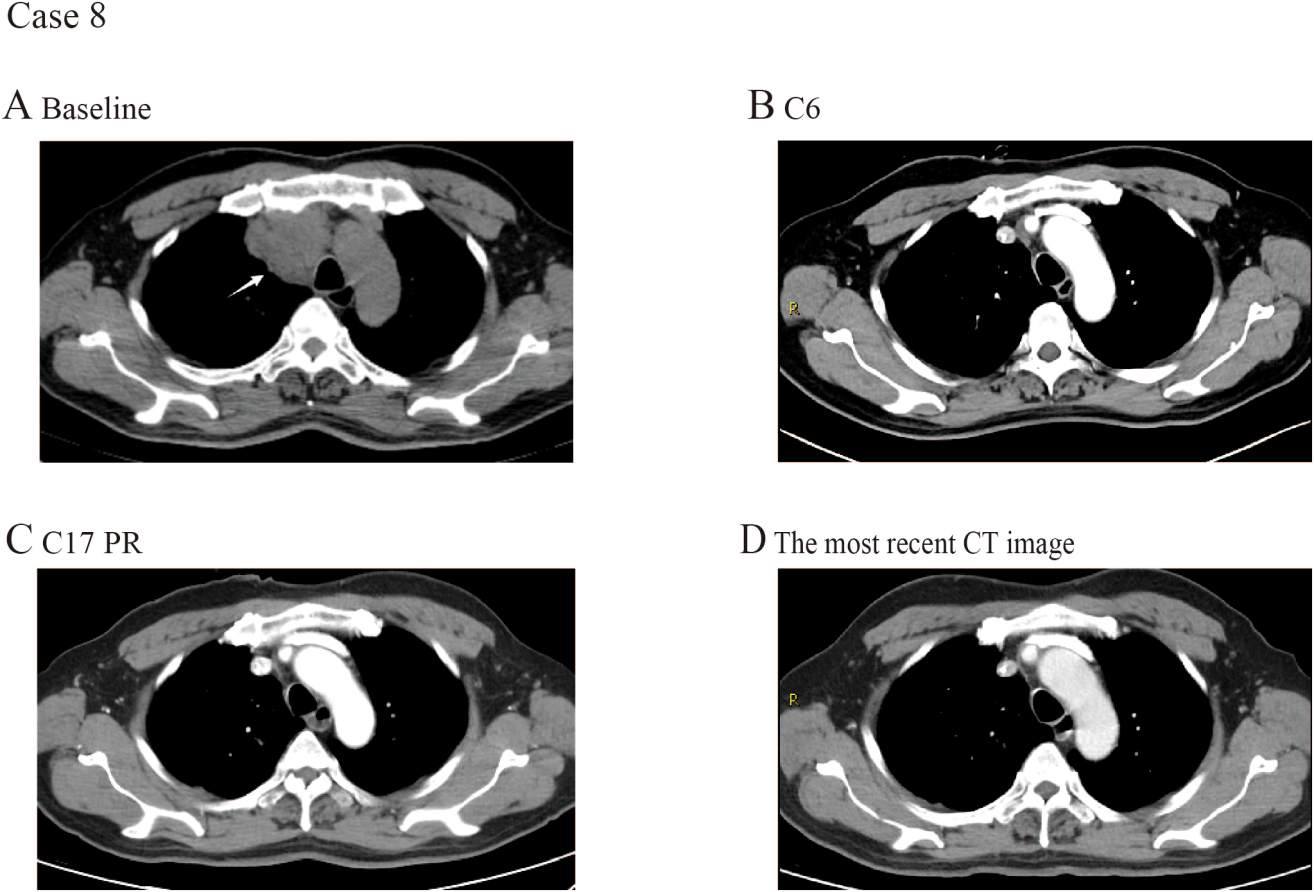
CT images selected from case 8. **(A)** CT scan examination of the primary tumor at the first time of diagnosis. **(B)** The primary tumor size decreased after 6 cycles of treatment. **(C)** The primary tumor size decreased after 17 cycles of treatment. **(D)** The most recent CT image.

A 58-year-old male arrived at the hospital presenting with hoarseness and was later diagnosed with stage IV TC. A CT scan disclosed a soft tissue mass measuring 5.2×3.6 cm in the right anterior upper mediastinum (**Figure 3A**). The PD-L1 expression level was not measured. Prior to his admission to our hospital, the patient had undergone three cycles of first-line treatment combining tislelizumab with chemotherapy from March 2023 to June 2023, during which a CT scan showed a reduction in tumor size. Given the favorable response observed, the patient continued to receive 3 cycles of tislelizumab combined with chemotherapy. Each 3-week cycle consisted of tislelizumab (200 mg), paclitaxel and carboplatin on Day 1. The primary tumor size decreased after 6 cycles of treatment (**Figure 3B**). The therapy was changed to maintenance therapy with tislelizumab alone at a dosage of 200 mg every 3 weeks from October 2022. After 8 cycles of monotherapy, a PR was observed, with the tumor further shrinking to 1.3×0.6 cm. The CT scan after 17 cycles of treatment showed that the tumor further shrinking to 1.2×0.5 cm (**Figure 3C**). Clinical evaluations and radiological assessments indicated no evidence of progression or recurrence, with the patient maintaining durable disease control for over 28 months as of the most recent follow-up in April 2024 (**Figure 3D**). Throughout the treatment course, the patient remained in good health and no immune-related adverse events occurred.

## Discussion

Thymic epithelial tumors (TETs) originate from thymic epithelial cells and can display varying levels of non-tumor immature lymphocytic infiltration (4). Although they are relatively rare and associated with poor prognoses, TETs are the most frequently occurring tumors in the anterior mediastinum (34). Many patients are diagnosed at advanced stages and face a lack of effective treatment options. The 5-year overall survival (OS) rates are approximately 90% for thymoma and 55% for thymic carcinoma (35, 36). Compared to thymic carcinoma, thymomas are often linked to autoimmune diseases because of the reduced expression of autoimmune regulator (AIRE) genes, major histocompatibility complex (MHC) molecules and the altered thymic architecture (37–40). Platinum-based chemotherapy is the standard first‐line treatment for patients with advanced TETs, but its efficacy is often suboptimal (41). While targeted therapies, such as anti-angiogenic kinase inhibitors, appear somewhat effective, the absence of actionable genomic alterations in TETs has hindered their further development (42).

Currently, immunotherapy is primarily utilized in the second-line treatment setting for TETs, with single-agent therapies being the predominant strategy (43). A phase II clinical trial by Cho et al. enrolled 33 patients (7 patients with T and 26 patients with TC) to evaluate the efficacy of pembrolizumab. The results indicated an overall response rate (ORR) of 21.2%, a disease control rate (DCR) of 78.8% and a median progression-free survival (PFS) of 6.1 months. Notably, irAEs were reported in 15.4% of thymic carcinoma patients, compared to a higher incidence of 71.6% among thymoma patients (20). In an another phase II study involving 40 patients with recurrent TC who had progressed after at least one cycle of chemotherapy, pembrolizumab was administered, resulting in severe irAEs in 15% of participants (23). This study noted an objective response rate of 22.5% and a disease progression rate of 25%. Moreover, the median PFS for patients with high PD-L1 expression reached 4.2 months, which significantly exceeded the 2.9 months median PFS observed in the low PD-L1 expression group (21). These findings corroborate observations from Cho et al., indicating that patients with higher PD-L1 levels tend to respond more favorably to immunotherapy.

Actually, the efficacy and safety of different immune checkpoint inhibitors (ICIs) vary significantly (44, 45). For instance, a phase II trial investigating the PD-1 inhibitor Nivolumab did not reveal significant responses among thymic carcinoma patients. Similar concerns regarding the risk of autoimmune disorders have been noted in other studies involving ICIs. Rajan et al. assessed the therapeutic impact of Avelumab in eight patients, but treatment was ultimately halted due to severe irAEs. These outcomes highlight the need for further investigation into the optimization of immunotherapy approaches for TETs (22, 46).

TETs are unique compared to other tumors due to their relatively high incidence of irAEs. Research indicates that the incidence of severe and fatal immune-mediated toxicity associated with ICBs in patients with thymoma ranges from 38% to 71.4%, whereas it is only 9% to 21% in patients with non-small cell lung cancer and melanoma receiving single-agent ICIs (22, 47, 48). The incidence of irAEs in TETs is observed to differ across various pathological stages (47). Research suggests a noteworthy trend where patients with B1/B2 and AB-type thymomas experience a higher frequency of irAEs compared to those with B3-type thymomas (49). This pattern underscores the complexity of immune responses in TETs and their varying interactions with treatment. Interestingly, the occurrence of irAEs often accompanies immune responses. For instance, Giaccone et al. reported that almost 50% of patients who suffered from severe irAEs attained partial responses, a rate that was considerably greater than that observed in patients without these adverse events (21). This phenomenon may result from disruptions in immune homeostasis, which can lead to systemic effects influenced by changes in immune cell diversity. Balancing efficacy and safety presents an ongoing challenge. Presently, research primarily focuses on investigating biomarkers associated with ICIs efficacy, with limited approaches to predict irAEs. Radiomic signature demonstrated potential in forecasting clinical outcomes, including the likelihood of irAEs, especially pneumonitis (50) Additionally, early alterations in B cell populations following combination checkpoint blockade may serve as indicators for identifying patients at high risk of irAEs (51). TETs often observe unique irAEs, such as muscle weakness, myocarditis and myositis, which are infrequently seen in other tumor types (21, 46). According to our previous study, some germline variants can associate with irAE and genetic testing might help guide the readministration of ICIs after initial irAEs in TETs (52). Further investigations are needed on the management of irAEs (53).

There are several ongoing studies about combination therapy using ICIs. Notably, Yuki Katsuya et al. observed that after chemotherapy, 30 patients exhibited a significant rise in PD-L1 expression, suggesting that combining PD-1/PD-L1 inhibitors or employing sequential therapy may lead to a more comprehensive treatment effect (54). The understanding of chemotherapy combined with immunotherapy for TETs is primarily derived from retrospective studies and case reports. A real-world retrospective study indicated that while the specific type of ICIs was not identified, the combination of therapies resulted in a marked improvement in PFS from 4.9 months to 8.7 months and increased ORR from 34% to 50%, compared to platinum-based chemotherapy alone, indicating a substantial response without an increase in toxicity (55).

Previous success outcomes inspired us to treat our patients with immunotherapy. Tislelizumab is a monoclonal antibody known for its high affinity for PD-1 (25). Clinical studies have indicated that standard doses of tislelizumab can be combined safely and effectively with chemotherapy across a range of solid tumors (28, 29). Chemotherapy can induce immunogenic cell death (ICD) in tumor cells, releasing tumor-specific antigens and enhancing the immune system’s ability to recognize and attack tumors. Chemotherapy also reduces immunosuppressive factors in the tumor micro-environment, such as regulatory T cells and tumor-associated macrophages, thereby improving the efficacy of immunotherapy. Furthermore, immunotherapy enhances the body’s antitumor immune response, making tumor cells more susceptible to chemotherapy. Taking the classic RATIONALE-307 study on tislelizumab as an example, combining tislelizumab with chemotherapy significantly prolonged median PFS compared to chemotherapy alone in patients with squamous NSCLC, irrespective of their PD-L1 expression levels (26). Despite these encouraging results, the rarity of TETs has led to a notable absence of clinical trials specifically assessing the safety and efficacy of tislelizumab in this patient population.

**Figure 4:**
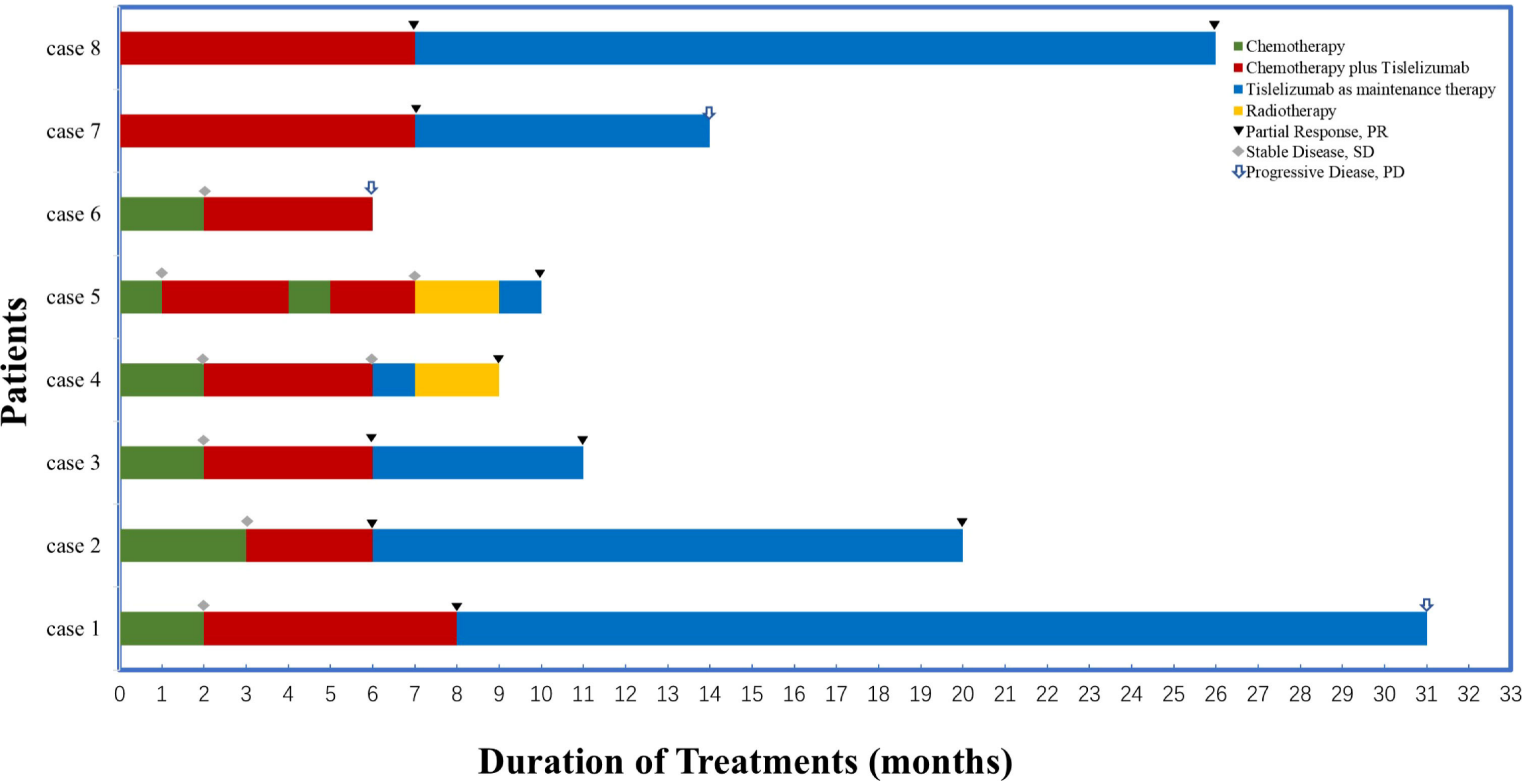
Timeline of the disease course and tumor responses. Response was assessed with response evaluation criteria in solid tumors version 1.1 by investigators.

All eight patients (seven with TC and one with T) were free of autoimmune disorder at baseline, especially no myasthenia gravis. Of the eight patients, six received immunotherapy as an adjunct to the initial chemotherapy can significantly enhance short-term effectiveness. The remaining two patients received initial treatment with tislelizumab plus chemotherapy and both achieved a partial clinical response during immunotherapy (**Figure 4**). The objective response rate (ORR) for this combination treatment was as high as 62.5%. Limited by follow-up time, we could not estimate the median survival for this cohort. cohort. All patients remained alive at the conclusion of the follow-up period. The longest PFS was 31 months, which suggests that the combination regimen has short-term efficacy and promising long-term efficacy. We observed a pseudo progression followed by partial response and subsequently progressive disease in this patient, it highlights the intricate nature of thymic immune tumors and warrants further exploration.

Interestingly, five of the eight patients in our case series tested negative for PD-L1 expression, yet we observed favorable disease control in this group. Four cases with negative PD-L1 expression exhibited more than ten months of PFS. Our results stand in contrast to previous reports indicating that patients with low PD-L1 expression in TETs did not exhibit significant responses (20). Nevertheless, in multiple large phase III studies of advanced non-small cell lung cancer, immunotherapy combined with chemotherapy demonstrated superior efficacy compared to PD-(L)1 inhibitor monotherapy, regardless of PD-L1 expression levels. This combination provides survival benefits across all patient subgroups and significantly reduces the risk of hyperprogression (56). For patients with high tumor burden, rapid progression, or severe symptoms, immunotherapy combined with chemotherapy can rapidly reduce tumor burden, alleviate symptoms, and improve survival outcomes, provided the patient can tolerate chemotherapy (50, 57). Our data further support the hypothesis that the selection and timing of immunotherapy may enhance efficacy. Tislelizumab combined with chemotherapy could represent a promising treatment strategy for patients with low PD-L1 expression (30–33).

Additionally, Toxicity is a major consideration for a key clinical decision. In our cases, combination therapy was well-tolerated. There were no immune-related grade 3–5 AEs, and all AEs were manageable with supportive measures. Grade 1–2 AEs were adrenal insufficiency (n=1), thyroid dysfunction (n=1), and pneumonia (n=1). The low incidence of irAEs in our case series might be attributable to following reasons. The first is the selection of patients. In our case series, there was only one case with thymoma, and irAEs seem more common in thymoma compared to thymic carcinoma (20). The second possibility is the selection of ICI, tislelizumab was purposefully designed to reduce its binding affinity to Fc-γ receptors (FcγRs) on macrophages, thereby minimizing the risk of antibody-dependent phagocytosis. This unique characteristic may result in a more favorable safety profile when compared to other immunotherapeutic agents. The third possibility is the selection of combining immunotherapy and chemotherapy. Increasing evidence indicates that this combination, particularly as a first-line therapy for various tumors, can lead to lower rates of most irAEs compared to the use of PD-(L)1 inhibitors alone (58).

While intriguing, our findings are constrained by the retrospective nature of the studies. We were unable to validate the PD-L1 expression levels for tislelizumab and the correlation with treatment efficacy. Furthermore, we did not conduct a comprehensive genomic analysis of the tumor samples. The next step is to investigate predictors of the patient population that would benefit from immunotherapy. Negative PD-L1 expression and successful treatment outcomes remind us to explore other features of the immune microenvironment, such as tumor mutation burden (TMB), microsatellite instability (MSI), and tumor-infiltrating T lymphocytes (TILs) (34, 59, 60). Therefore, we need to gather more data on the immune microenvironment of TETs. Additionally, a prospective, single-arm trial evaluating tislelizumab in combination with standard chemotherapy as a first-line treatment in advanced TETs is currently underway.

In summary, our case series explores the feasibility of tislelizumab in advanced TETs within a real-world clinical context, demonstrating preliminary evidence for the efficacy and safety of tislelizumab combined with chemotherapy followed by maintenance monotherapy. Notably, while current clinical trials predominantly focus on thymic carcinoma populations, this series includes a thymoma case, providing initial insights into the potential applicability of tislelizumab in thymoma management and expanding the therapeutic landscape for TETs beyond thymic carcinoma-specific studies.

## Conclusion

The combination of tislelizumab and chemotherapy may serve as a promising approach for the initial or even first-line management of advanced TETs, thereby necessitating additional investigation. The favorable results noted in most of our patients with low PD-L1 expression indicate that the application of chemo-immunotherapy may not be restricted to individuals with PD-L1 levels exceeding 50%. Ongoing research is also focused on examining biomarkers to identify potential populations that could benefit from this treatment.

## Data Availability

The original contributions presented in the study are included in the article/**Supplementary Material**. Further inquiries can be directed to the corresponding authors.

## References

[1] Harnath T Marx A Ströbel P Bölke E Willers R Gripp S. Thymoma-a clinico-pathological long-term study with emphasis on histology and adjuvant radiotherapy dose. J thoracic oncology: Off Publ Int Assoc Study Lung Cancer. (2012) 7:1867–71. doi: 10.1097/JTO.0b013e3182745f73 23154559

[2] De Jong WK Blaauwgeers JL Schaapveld M Timens W Klinkenberg TJ Groen HJ. Thymic epithelial tumours: a population-based study of the incidence, diagnostic procedures and therapy. Eur J Cancer (Oxford England: 1990). (2008) 44:123–30. doi: 10.1016/j.ejca.2007.11.004 18068351

[3] Xu C Zhang Y Wang W Wang Q Li Z Song Z. Chinese expert consensus on the diagnosis and treatment of thymic epithelial tumors. Thoracic Cancer. (2023) 14:1102–17. doi: 10.1111/1759-7714.14847 PMC10125784 36924056

[4] Marx A Chan JKC Chalabreysse L Dacic S Detterbeck F French CA. The 2021 WHO classification of tumors of the thymus and mediastinum: what is new in thymic epithelial, germ cell, and mesenchymal tumors? J Thorac Oncol. (2022) 17:200–13. doi: 10.1016/j.jtho.2021.10.010 34695605

[5] Ströbel P Weis CA Marx A. Thymic carcinomas. Pathologe. (2016) 37:425–33. doi: 10.1007/s00292-016-0194-4 27538748

[6] Berghmans T Durieux V Holbrechts S Jungels C Lafitte JJ Meert AP. Systemic treatments for thymoma and thymic carcinoma: A systematic review. Lung Cancer (Amsterdam Netherlands). (2018) 126:25–31. doi: 10.1016/j.lungcan.2018.10.018 30527189

[7] Merveilleux Du Vignaux C Dansin E Mhanna L Greillier L Pichon E Kerjouan M. Systemic therapy in advanced thymic epithelial Tumors: insights from the RYTHMIC prospective cohort. J thoracic oncology: Off Publ Int Assoc Study Lung Cancer. (2018) 13:1762–70. doi: 10.1016/j.jtho.2018.08.005 30138763

[8] Furugen M Sekine I Tsuta K Horinouchi H Nokihara H Yamamoto N. Combination chemotherapy with carboplatin and paclitaxel for advanced thymic cancer. Japanese J Clin Oncol. (2011) 41:1013–6. doi: 10.1093/jjco/hyr089 21742653

[9] Hirai F Yamanaka T Taguchi K Daga H Ono A Tanaka K. A multicenter phase II study of carboplatin and paclitaxel for advanced thymic carcinoma: WJOG4207L. Ann oncology: Off J Eur Soc Med Oncol. (2015) 26:363–8. doi: 10.1093/annonc/mdu541 25403584

[10] Igawa S Murakami H Takahashi T Nakamura Y Tsuya A Naito T. Efficacy of chemotherapy with carboplatin and paclitaxel for unresectable thymic carcinoma. Lung Cancer (Amsterdam Netherlands). (2010) 67:194–7. doi: 10.1016/j.lungcan.2009.03.031 19409644

[11] Lemma GL Lee JW Aisner SC Langer CJ Tester WJ Johnson DH. Phase II study of carboplatin and paclitaxel in advanced thymoma and thymic carcinoma. J Clin oncology: Off J Am Soc Clin Oncol. (2011) 29:2060–5. doi: 10.1200/JCO.2010.32.9607 PMC3107762 21502559

[12] Scorsetti M Leo F Trama A D'angelillo R Serpico D Macerelli M. Thymoma and thymic carcinomas. Crit Rev oncology/hematology. (2016) 99:332–50. doi: 10.1016/j.critrevonc.2016.01.012 26818050

[13] Manti PG Trattaro S Castaldi D Pezzali M Spaggiari L Testa G. Thymic stroma and TFII-I: towards new targeted therapies. Trends Mol Med. (2022) 28:67–78. doi: 10.1016/j.molmed.2021.10.008 34865984

[14] Merveilleux Du Vignaux C Maury JM Girard N. Novel agents in the treatment of thymic Malignancies. Curr Treat options Oncol. (2017) 18:52. doi: 10.1007/s11864-017-0495-8 28795288

[15] Weissferdt A Fujimoto J Kalhor N Rodriguez J Bassett R Wistuba I. Expression of PD-1 and PD-L1 in thymic epithelial neoplasms. Modern pathology: an Off J United States Can Acad Pathology Inc. (2017) 30:826–33. doi: 10.1038/modpathol.2017.6 28281549

[16] Marchevsky AM Walts AE. PD-L1, PD-1, CD4, and CD8 expression in neoplastic and nonneoplastic thymus. Hum Pathol. (2017) 60:16–23. doi: 10.1016/j.humpath.2016.09.023 27746267

[17] Katsuya Y Fujita Y Horinouchi H Ohe Y Watanabe S Tsuta K. Immunohistochemical status of PD-L1 in thymoma and thymic carcinoma. Lung Cancer (Amsterdam Netherlands). (2015) 88:154–9. doi: 10.1016/j.lungcan.2015.03.003 25799277

[18] Padda SK Riess JW Schwartz EJ Tian L Kohrt HE Neal JW. Diffuse high intensity PD-L1 staining in thymic epithelial tumors. J thoracic oncology: Off Publ Int Assoc Study Lung Cancer. (2015) 10:500–8. doi: 10.1097/JTO.0000000000000429 PMC4720257 25402569

[19] Berardi R Goteri G Brunelli A Pagliaretta S Paolucci V Caramanti M. Prognostic relevance of programmed cell death protein 1/programmed death-ligand 1 pathway in thymic Malignancies with combined immunohistochemical and biomolecular approach. Expert Opin Ther Targets. (2020) 24:937–43. doi: 10.1080/14728222.2020.1790529 32662701

[20] Cho J Kim HS Ku BM Choi YL Cristescu R Han J. Pembrolizumab for patients with refractory or relapsed thymic epithelial tumor: an open-label phase II trial. J Clin oncology: Off J Am Soc Clin Oncol. (2019) 37:2162–70. doi: 10.1200/JCO.2017.77.3184 29906252

[21] Giaccone G Kim C Thompson J Mcguire C Kallakury B Chahine JJ. Pembrolizumab in patients with thymic carcinoma: a single-arm, single-centre, phase 2 study. Lancet Oncol. (2018) 19:347–55. doi: 10.1016/S1470-2045(18)30062-7 PMC10683856 29395863

[22] Rajan A Heery CR Thomas A Mammen AL Perry S O'sullivan Coyne G. Efficacy and tolerability of anti-programmed death-ligand 1 (PD-L1) antibody (Avelumab) treatment in advanced thymoma. J immunotherapy Cancer. (2019) 7:269. doi: 10.1186/s40425-019-0723-9 PMC6805423 31639039

[23] Mammen AL Rajan A Pak K Lehky T Casciola-Rosen L Donahue RN. Pre-existing antiacetylcholine receptor autoantibodies and B cell lymphopaenia are associated with the development of myositis in patients with thymoma treated with avelumab, an immune checkpoint inhibitor targeting programmed death-ligand 1. Ann rheumatic Dis. (2019) 78:150–2. doi: 10.1136/annrheumdis-2018-213777 PMC6447048 30185415

[24] NCCN Guidelines Version 1.2022. Thymomas and Thymic Carcinomas. (America: National Comprehensive Cancer Network). (2022). Available online at: https://www.nccn.org/guidelines/guidelines-detail?category=1&id=1469.

[25] Hong Y Feng Y Sun H Zhang B Wu H Zhu Q. Tislelizumab uniquely binds to the CC' loop of PD-1 with slow-dissociated rate and complete PD-L1 blockage. FEBS Open Bio. (2021) 11:782–92. doi: 10.1002/2211-5463.13102 PMC7931243 33527708

[26] Wang J Lu S Yu X Hu Y Sun Y Wang Z. Tislelizumab plus chemotherapy vs chemotherapy alone as first-line treatment for advanced squamous non-small-cell lung cancer: A phase 3 randomized clinical trial. JAMA Oncol. (2021) 7:709–17. doi: 10.1001/jamaoncol.2021.0366 PMC8017481 33792623

[27] Osarogiagbon RU. Tislelizumab-A promising new option for enhancing chemotherapy benefit in treatment for advanced squamous cell lung cancer. JAMA Oncol. (2021) 7:717–9. doi: 10.1001/jamaoncol.2021.0262 33792622

[28] Shen L Kato K Kim SB Ajani JA Zhao K He Z. Tislelizumab versus chemotherapy as second-line treatment for advanced or metastatic esophageal squamous cell carcinoma (RATIONALE-302): A randomized phase III study. J Clin oncology: Off J Am Soc Clin Oncol. (2022) 40:3065–76. doi: 10.1200/JCO.21.01926 PMC9462531 35442766

[29] Zhou C Huang D Fan Y Yu X Liu Y Shu Y. Tislelizumab versus docetaxel in patients with Previously treated advanced NSCLC (RATIONALE-303): A phase 3, open-label, randomized controlled trial. J thoracic oncology: Off Publ Int Assoc Study Lung Cancer. (2023) 18:93–105. doi: 10.1016/j.jtho.2022.09.217 36184068

[30] Zhou C Chen G Huang Y Zhou J Lin L Feng J. Camrelizumab plus carboplatin and pemetrexed versus chemotherapy alone in chemotherapy-naive patients with advanced non-squamous non-small-cell lung cancer (CameL): a randomised, open-label, multicentre, phase 3 trial. Lancet Respiratory Med. (2021) 9:305–14. doi: 10.1016/S2213-2600(20)30365-9 33347829

[31] Yang Y Wang Z Fang J Yu Q Han B Cang S. Efficacy and Safety of Sintilimab Plus Pemetrexed and Platinum as First-Line Treatment for Locally Advanced or Metastatic Nonsquamous NSCLC: a Randomized, Double-Blind, Phase 3 Study (Oncology pRogram by InnovENT anti-PD-1-11). J thoracic oncology: Off Publ Int Assoc Study Lung Cancer. (2020) 15:1636–46. doi: 10.1016/j.jtho.2020.07.014 32781263

[32] Nishio M Barlesi F West H Ball S Bordoni R Cobo M. Atezolizumab plus chemotherapy for first-line treatment of nonsquamous NSCLC: results from the randomized phase 3 IMpower132 trial. J thoracic oncology: Off Publ Int Assoc Study Lung Cancer. (2021) 16:653–64. doi: 10.1016/j.jtho.2020.11.025 33333328

[33] Matsuo N Azuma K Kojima T Ishii H Tokito T Yamada K. Comparative incidence of immune-related adverse events and hyperprogressive disease in patients with non-small cell lung cancer receiving immune checkpoint inhibitors with and without chemotherapy. Investigational New Drugs. (2021) 39:1150–8. doi: 10.1007/s10637-021-01069-7 33483882

[34] Conforti F Pala L Giaccone G De Pas T. Thymic epithelial tumors: From biology to treatment. Cancer Treat Rev. (2020) 86:102014. doi: 10.1016/j.ctrv.2020.102014 32272379

[35] Masaoka A. Staging system of thymoma. J thoracic oncology: Off Publ Int Assoc Study Lung Cancer. (2010) 5:S304–12. doi: 10.1097/JTO.0b013e3181f20c05 20859124

[36] Weksler B Dhupar R Parikh V Nason KS Pennathur A Ferson PF. Thymic carcinoma: a multivariate analysis of factors predictive of survival in 290 patients. Ann thoracic Surg. (2013) 95:299–303. doi: 10.1016/j.athoracsur.2012.09.006 23141529

[37] Marx A Hohenberger P Hoffmann H Pfannschmidt J Schnabel P Hofmann HS. The autoimmune regulator AIRE in thymoma biology: autoimmunity and beyond. J thoracic oncology: Off Publ Int Assoc Study Lung Cancer. (2010) 5:S266–72. doi: 10.1097/JTO.0b013e3181f1f63f 20859117

[38] Burbelo PD Browne SK Sampaio EP Giaccone G Zaman R Kristosturyan E. Anti-cytokine autoantibodies are associated with opportunistic infection in patients with thymic neoplasia. Blood. (2010) 116:4848–58. doi: 10.1182/blood-2010-05-286161 PMC3321746 20716769

[39] Saito M Fujiwara Y Asao T Honda T Shimada Y Kanai Y. The genomic and epigenomic landscape in thymic carcinoma. Carcinogenesis. (2017) 38:1084–91. doi: 10.1093/carcin/bgx094 28968686

[40] Ao YQ Gao J Wang S Jiang JH Deng J Wang HK. Immunotherapy of thymic epithelial tumors: molecular understandings and clinical perspectives. Mol Cancer. (2023) 22:70. doi: 10.1186/s12943-023-01772-4 37055838 PMC10099901

[41] Roden AC Ahmad U Cardillo G Girard N Jain D Marom EM. Thymic carcinomas-A concise multidisciplinary update on recent developments from the thymic carcinoma working group of the international thymic Malignancy interest group. J thoracic oncology: Off Publ Int Assoc Study Lung Cancer. (2022) 17:637–50. doi: 10.1016/j.jtho.2022.01.021 PMC11080660 35227908

[42] Radovich M Pickering CR Felau I Ha G Zhang H Jo H. The integrated genomic landscape of thymic epithelial tumors. Cancer Cell. (2018) 33:244–58.e10. doi: 10.1016/j.ccell.2018.01.003 29438696 PMC5994906

[43] Remon J Passiglia F Ahn MJ Barlesi F Forde PM Garon EB. Immune checkpoint inhibitors in thoracic Malignancies: review of the existing evidence by an IASLC expert panel and recommendations. J thoracic oncology: Off Publ Int Assoc Study Lung Cancer. (2020) 15:914–47. doi: 10.1016/j.jtho.2020.03.006 32179179

[44] Conforti F Zucali PA Pala L Catania C Bagnardi V Sala I. Avelumab plus axitinib in unresectable or metastatic type B3 thymomas and thymic carcinomas (CAVEATT): a single-arm, multicentre, phase 2 trial. Lancet Oncol. (2022) 23:1287–96. doi: 10.1016/S1470-2045(22)00542-3 36096156

[45] Fenioux C Abbar B Boussouar S Bretagne M Power JR Moslehi JJ. Thymus alterations and susceptibility to immune checkpoint inhibitor myocarditis. Nat Med. (2023) 29:3100–10. doi: 10.1038/s41591-023-02591-2 37884625

[46] Katsuya Y Horinouchi H Seto T Umemura S Hosomi Y Satouchi M. Single-arm, multicentre, phase II trial of nivolumab for unresectable or recurrent thymic carcinoma: PRIMER study. Eur J Cancer (Oxford England: 1990). (2019) 113:78–86. doi: 10.1016/j.ejca.2019.03.012 30991261

[47] Maniar R Loehrer PJSR. Understanding the landscape of immunotherapy in thymic epithelial tumors. Cancer. (2023) 129:1162–72. doi: 10.1002/cncr.v129.8 36808725

[48] Reck M Rodríguez-Abreu D Robinson AG Hui R Csőszi T Fülöp A. Pembrolizumab versus chemotherapy for PD-L1-positive non-small-cell lung cancer. New Engl J Med. (2016) 375:1823–33. doi: 10.1056/NEJMoa1606774 27718847

[49] Hao Y Lin G Xiang J Wang W Xu C Wang Q. Analysis of the efficacy and safety of immunotherapy in advanced thymoma patients. Cancer Med. (2023) 12:5649–55. doi: 10.1002/cam4.v12.5 PMC10028091 36394097

[50] Paz-Ares L Vicente D Tafreshi A Robinson A Soto Parra H Mazières J. A randomized, placebo-controlled trial of pembrolizumab plus chemotherapy in patients with metastatic squamous NSCLC: protocol-specified final analysis of KEYNOTE-407. J thoracic oncology: Off Publ Int Assoc Study Lung Cancer. (2020) 15:1657–69. doi: 10.1016/j.jtho.2020.06.015 32599071

[51] Das R Bar N Ferreira M Newman AM Zhang L Bailur JK. Early B cell changes predict autoimmunity following combination immune checkpoint blockade. J Clin Invest. (2018) 128:715–20. doi: 10.1172/JCI96798 PMC5785243 29309048

[52] Gao W Wu L Jin S Li J Liu X Xu J. Rechallenge of immune checkpoint inhibitors in a case with adverse events inducing myasthenia gravis. J immunotherapy Cancer. (2022) 10. doi: 10.1136/jitc-2022-005970 PMC9716945 36450378

[53] Rajan A. Immunotherapy for thymic cancers: A convoluted path toward a cherished goal. J thoracic oncology: Off Publ Int Assoc Study Lung Cancer. (2021) 16:352–4. doi: 10.1016/j.jtho.2020.12.007 33641717

[54] Katsuya Y Horinouchi H Asao T Kitahara S Goto Y Kanda S. Expression of programmed death 1 (PD-1) and its ligand (PD-L1) in thymic epithelial tumors: Impact on treatment efficacy and alteration in expression after chemotherapy. Lung Cancer (Amsterdam Netherlands). (2016) 99:4–10. doi: 10.1016/j.lungcan.2016.05.007 27565906

[55] Liu M Zhao J Liu Z Hou X. MA08.08 First-line PD-1 Inhibitor plus Chemotherapy versus Platinum-based Chemotherapy for Patients with Advanced Thymic Carcinoma. J Thorac Oncol. (2023) 18:S127. doi: 10.1016/j.jtho.2023.09.169

[56] Gandhi L Rodríguez-Abreu D Gadgeel S Esteban E Felip E De Angelis F. Pembrolizumab plus chemotherapy in metastatic non-small-cell lung cancer. New Engl J Med. (2018) 378:2078–92. doi: 10.1056/NEJMoa1801005 29658856

[57] Gadgeel S Rodríguez-Abreu D Speranza G Esteban E Felip E Dómine M. Updated analysis from KEYNOTE-189: pembrolizumab or placebo plus pemetrexed and platinum for previously untreated metastatic nonsquamous non-small-cell lung cancer. J Clin oncology: Off J Am Soc Clin Oncol. (2020) 38:1505–17. doi: 10.1200/JCO.19.03136 32150489

[58] Wang M Liang H Wang W Zhao S Cai X Zhao Y. Immune-related adverse events of a PD-L1 inhibitor plus chemotherapy versus a PD-L1 inhibitor alone in first-line treatment for advanced non-small cell lung cancer: A meta-analysis of randomized control trials. Cancer. (2021) 127:777–86. doi: 10.1002/cncr.v127.5 33119182

[59] Alexandrov LB Nik-Zainal S Wedge DC Aparicio SA Behjati S Biankin AV. Signatures of mutational processes in human cancer. Nature. (2013) 500:415–21. doi: 10.1038/nature12477 PMC3776390 23945592

[60] Zhang X Zhang P Cong A Feng Y Chi H Xia Z. Unraveling molecular networks in thymic epithelial tumors: deciphering the unique signatures. Front Immunol. (2023) 14:1264325. doi: 10.3389/fimmu.2023.1264325 37849766 PMC10577431

